# How Many Anxious Kids in Community Mental Health Would Be Eligible for an RCT? And Does It Matter? Insights from a Naturalistic Sample and a Non-Systematic Review

**DOI:** 10.3390/children13030413

**Published:** 2026-03-18

**Authors:** Anya C. English, Megan Brady, Amanda L. Sanchez, Emily M. Becker-Haimes

**Affiliations:** 1 Department of Psychiatry, University of Pennsylvania, Philadelphia, PA 19104, USA; anya.english@pennmedicine.upenn.edu (A.C.E.); megan.brady@pennmedicine.upenn.edu (M.B.); 2 Department of Psychology, George Mason University, Fairfax, VA 22030, USA; asanch49@gmu.edu; 3 Hall Mercer Community Mental Health, University of Pennsylvania Health System, Philadelphia, PA 19104, USA

**Keywords:** randomized controlled trials, exposure therapy, pediatric, anxiety disorders, effectiveness

## Abstract

**Highlights:**

**What are the main findings?**

- Among youth seeking specialty anxiety treatment in a community mental health setting, the majority would have been ineligible for at least one published randomized controlled trial of cognitive behavioral therapy for pediatric anxiety and related disorders.
- Ineligible youth required nearly twice as many treatment sessions and were more than twice as likely to receive case management services.

**What is the implication of the main finding?**

- These results do not imply cognitive behavioral therapy is less effective for clinically complex youth. Rather, they suggest that youth commonly excluded from efficacy trials may require more flexible or prolonged care than is typically represented in tightly controlled RCT protocols.
- Trial exclusion status may potentially serve as a prognostic indicator in community settings, helping clinicians set realistic expectations for families and signal when treatment adaptation or added supports are warranted.

**Abstract:**

**Background**: Decades of randomized controlled trials (RCTs) support cognitive behavioral therapy (CBT) for pediatric anxiety, but exclusion criteria may limit generalizability to routine settings. We examined common exclusion criteria in recent CBT RCTs for pediatric anxiety, trends in these criteria over time, and whether meeting RCT exclusion criteria affects outcomes in a naturalistic sample. **Methods**: We reviewed 81 RCTs from the past 25 years assessing CBT for pediatric anxiety or related disorders to identify common exclusion criteria. We examined how often youth seeking exposure-based treatment for anxiety or OCD at an urban community health center met these exclusion criteria and whether this impacted treatment response, using three-year retrospective chart review data (*n* = 94). **Results**: Common exclusion criteria in identified RCTs included psychotropic medication use (66.7%), autism spectrum disorder (63.0%), and other psychiatric comorbidities. Suicidal ideation increased as an exclusion criterion over time (*p* < 0.05, Cramér’s *V* = 0.23). Based on these criteria, 53% of participants in our naturalistic sample would have been excluded from one or more RCTs. Excluded patients did not differ in baseline characteristics. Excluded youth required nearly twice as many treatment sessions and had more than double the rate of case management utilization (all *p*s < 0.01). **Conclusions**: Youth who would have been excluded from at least one RCT had poorer prognoses. Findings support continued emphasis on pragmatic trials to advance understanding of how to augment treatments to better meet the diverse needs of youth.

## 1. Introduction

Cognitive behavioral therapy (CBT) is the most widely used and well-supported intervention for treating anxiety and related disorders in children [1]. Decades of research and numerous randomized controlled trials (RCTs) have established CBT’s efficacy and effectiveness, particularly for generalized anxiety disorder (GAD), social anxiety disorder (SAD), obsessive–compulsive disorder (OCD), panic disorder, and specific phobias [2,3,4,5]. As a result, CBT is considered a front-line treatment for youth suffering from anxiety and related disorders [6]. Across studies, CBT has outperformed wait-list controls, treatment as usual, and attention controls [7,8]. Efficacy trials have also shown that CBT-based interventions are superior to alternative methods such as relaxation, bibliotherapy, family-based support, and emotional disclosure in the treatment of childhood anxiety disorders [9,10,11,12,13].

RCTs are generally considered the most rigorous level of scientific evidence we have to evaluate the efficacy and effectiveness of psychotherapy [14]. Common to RCTs is the use of exclusion criteria, which are routinely employed in clinical research trials to isolate the effects of an evidence-based treatment (EBT) on target mechanisms of interest and minimize confounding variables, serving as a necessary component of rigorous science. For example, CBT efficacy trials for anxiety disorders may exclude certain comorbidities (e.g., depression, Attention-Deficit/Hyperactivity Disorder [ADHD]) or historical factors (e.g., past treatment history, certain medications) that would complicate evaluation of whether treatment was having the intended effects [15,16].

Despite the demonstrated efficacy of CBT for pediatric anxiety, there are concerns about how tight exclusion criteria commonly used in randomized controlled trials (RCTs) may limit the applicability of research findings to youth served in community mental health settings [17,18]. For example, Weisz et al. [19] found that across a multi-decade review of pediatric anxiety RCT samples, only 2.1% of patients had been clinically referred clients, who typically show higher symptom severity, greater comorbidity, and more complex treatment needs; these characteristics may contribute to the underrepresentation of clinically complex patients in RCTs [20,21]. Additionally, a prior study found that 60.8% of youth in a community mental health setting were actively using psychotropic medication, a cause for exclusion from many RCTs seeking to isolate the effect of CBT [22]. Many participants in community settings also frequently present with multiple comorbid diagnoses [23,24]. Taken together, this suggests that, perhaps in part due to exclusion criteria, the clinical profiles of treatment-seeking youth may contain greater complexity than those of youth who are treated within RCTs. To our knowledge, no studies to date have examined the trends in exclusion criteria for RCTs of CBT. While it is established that a substantial proportion of clinically complex youth would be excluded from efficacy trials, it remains unknown whether the scope of these exclusions has expanded or narrowed over time, which specific criteria are driving any change, and the implications this has for the applicability of CBT findings to real-world clinical populations.

Closely related to the issue of exclusion criteria is that of sample representativeness with respect to the sociodemographic and cultural background of treated individuals. Most early CBT efficacy research relied on largely socioeconomically and demographically homogeneous samples in Europe and the United States, with limited representation of racially and ethnically diverse and economically disadvantaged individuals [25]. In other disciplines, tight exclusion criteria also can lead to disproportionately low representation of individuals of color in clinical trials [26]; limited representation of communities of color within RCTs is also exacerbated by limited targeted outreach, physical inaccessibility of research institutions (where many trials take place), and historic injustices such as the Tuskegee Syphilis Study that leads to understandable hesitancy among communities of color to participate in clinical research [27,28]. Multiple studies similarly demonstrate that youth treated in routine clinical settings differ in clinical presentation and racial, ethnic, and socioeconomic backgrounds from those enrolled in research trials [23,24]. Collectively, these patterns highlight a significant gap in the literature: relatively little is known from gold-standard RCT clinical trials about how CBT functions for clinically complex youth and those from socioeconomically, racially, and ethnically diverse backgrounds.

When CBT has been tested outside the controlled conditions of efficacy trials, the research for its effectiveness for pediatric anxiety and related disorders is less consistent. Recent Cochrane reviews have questioned whether community-delivered CBT offers significant advantages over psychoeducation, bibliotherapy, treatment-as-usual, or other supportive interventions for pediatric anxiety [29]. Similarly, studies comparing CBT with selective serotonin reuptake inhibitors (SSRIs) in real-world settings show no clear superiority [30,31]. This pattern persists across treatment modalities, where school and community-based CBT programs often yield outcomes comparable to treatment-as-usual [8,32]. As study design moves from tightly controlled designs to more pragmatic trials, treatment effects tend to diminish, a phenomenon described as the “implementation cliff” [19]. The gap between CBT’s strong efficacy in RCTs and inconsistent effectiveness findings has prompted questions about how demographic and clinical differences between trial participants and community populations may affect the implementation of CBT. Much effort has been made in the past few decades to examine how CBT protocols can be best applied in routine clinical settings to meet the diverse needs of youth who may not fit the tight mold of an efficacy RCT. For example, modular treatment designs that allow clinicians to match treatment techniques to the presenting problems of youth with complex presentations can increase the effectiveness of CBT in routine clinical settings [33,34].

That said, persistent concerns remain about the “transportability” of RCT findings to routine practice, especially for anxiety-related disorders [35,36]. Despite growing recognition and study of the “implementation cliff” in youth anxiety treatment [19], no published study has examined how exclusion criteria contribute to implementation challenges, nor whether the clinical and demographic disparities between RCT samples and community populations, a partial consequence of study exclusion criteria, influence treatment effectiveness. This study addresses this gap directly, contributing to an emerging body of scholarship aimed at bridging the science-to-practice divide in evidence-based mental health care for youth. This study pursues three primary, exploratory objectives: (1) identify the exclusion criteria employed in recent RCTs of CBT for pediatric anxiety, (2) examine how the use of these criteria has evolved over time, and (3) examine the impact of youth meeting exclusion criteria on treatment outcomes. This final objective included two related research questions: (a) What proportion of treatment-seeking youth in a naturalistic treatment sample would have been excluded from published efficacy RCTs? and (b) were excluded youth distinguishable from eligible youth in terms of baseline clinical presentation and treatment outcomes? For this final aim, we leveraged data from a naturalistic sample of racially and economically diverse youth receiving exposure-based CBT (EX-CBT) at a specialty anxiety treatment program within an urban community mental health center in the Northeastern United States.

## 2. Method

### 2.1. Exclusion Criteria in Recent CBT RCTs

We conducted a narrative review of RCTs evaluating the efficacy of CBT for pediatric anxiety and related disorders, irrespective of study design, published between 2000 and 2024. A search was conducted on PubMed using the Advanced Search Builder. The terms “pediatric,” “youth,” “cognitive behavioral therapy,” and “meta-analysis” were combined using the AND operator across all fields, with “anxiety” OR “OCD” included to capture either condition. Results were filtered to studies published within the last decade. The final query was: (pediatric) AND (youth) AND (cognitive behavioral therapy) AND (meta-analysis) AND (anxiety OR OCD). The search yielded 40 articles, of which 30 were excluded because they did not focus on pediatric anxiety or a related disorder, did not employ a meta-analytic design, did not include randomized controlled trials (RCTs), or were duplicates. This resulted in a final sample of 10 meta-analyses. Individual RCTs were extracted through backward citation chaining of the included meta-analyses. Unlike a systematic review, this search was confined to a single database (PubMed) and was conducted without a pre-registered protocol.

**Figure 1:**
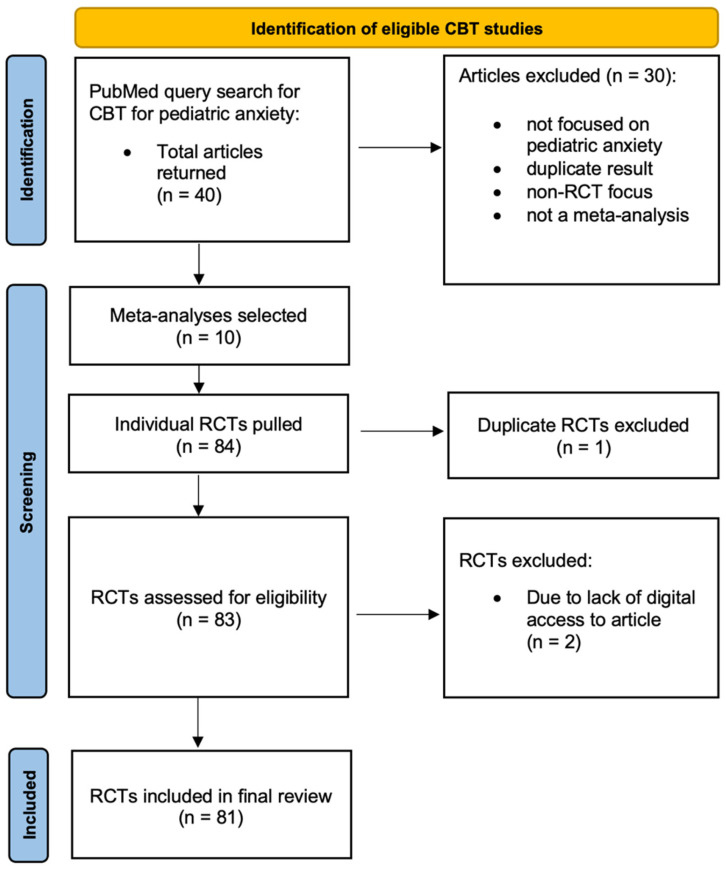
Flow diagram of RCT inclusion.

RCTs were eligible for inclusion in the literature review if they included participants aged 18 years or younger who were seeking treatment for a primary anxiety disorder or school-related anxiety. RCTs also had to include an intervention group testing some type of individualized CBT intervention that compared CBT to a waitlist control, treatment-as-usual, or another psychological treatment. Group-based interventions were excluded to maintain comparability with our naturalistic data comparison sample. Trials allowing psychotropic medication use were included only when individual-based CBT was evaluated in combination with medication (e.g., SSRIs + CBT). Studies comparing CBT solely to medication, or those that required all participants to take medication, were excluded. Effectiveness trials were also excluded to focus specifically on exclusion criteria used in tightly controlled efficacy studies, where originally developed treatment protocols are most often tested [37]. We included eligible trials if they met both the population and CBT intervention-comparison inclusion criteria. Studies from any country and in any language were eligible, provided they satisfied these criteria. Those not written in English were read via web-based translators. The lead author (AE) reviewed all studies to confirm study eligibility. Co-author (MB) independently reviewed 10% of identified studies to confirm eligibility with 100% agreement. In total, 81 studies met the inclusion criteria (see Figure 1).

Appendix A includes an overview of the characteristics of the reviewed RCTs. All sourced meta-analyses were published after 2015, with more than half of the included RCTs dating from 2012 or later. Most included RCTs were conducted in the United States (*n* = 42; 51.85%), followed by Europe (*n* = 27; 33.33%) and Australia (*n* = 10; 12.35%). The sample sizes within the RCTs ranged from 10 to 488 participants (median = 55), totaling 6405 individuals across the 81 studies. The age ranges of recruited samples across CBT studies were highly heterogeneous and specific to each study; this sample included children aged 3–18 years. Similarly, the included diagnoses varied greatly between studies, with the most frequent being social anxiety disorder (35.8%), specific phobia (29.6%), obsessive–compulsive disorder (28.4%), generalized anxiety disorder (24.7%), or simply any primary pediatric anxiety disorder (23.5%). CBT was delivered with family involvement in over one-quarter of the RCTs reviewed (*n* = 21).

RCT exclusion criteria varied significantly between studies. Criteria occurring less than four times (e.g., pregnancy, history of autoimmune disease) or described too vaguely to consistently track (e.g., generally poor physical health, parent suffering from severe psychiatric disorder) were not included in our analysis of RCT criteria. We identified six criteria that were employed in at least 10% of reviewed RCTs. These common RCT exclusion criteria included current psychotropic medication use, comorbid autism spectrum disorder (ASD) diagnosis, comorbid psychosis, suicidal ideation, comorbid major depressive disorder (MDD) diagnosis, and comorbid ADHD diagnosis. Each study was coded to include binary variables (‘yes’ or ‘no’) corresponding to the presence of each of these six exclusion criteria in each trial. These were summed descriptively.

### 2.2. Trends in Exclusion Criteria over Time

#### Statistical Analyses

RCT data were subset into three-decade groups: 2000–2009 (*n* = 22), 2010–2019 (*n* = 52), and 2020–2024 (*n* = 7). Because the 2020–2024 decade group had a small sample size, we used Freeman-Halton extensions of Fisher’s exact test within a 3 × 2 design to explore whether the prevalence of each exclusion criterion varied significantly across decades. Each Fisher’s exact test was evaluated at a conventional threshold of α = 0.05. Effect sizes were calculated using Cramér’s V.

### 2.3. Comparison to a Naturalistic Treatment Sample

We performed a retrospective, observational study of naturalistic data collected during routine clinical care. No experimental manipulation or randomization was conducted, and this investigation was not a clinical trial. This analysis is reported in accordance with the STROBE (Strengthening the Reporting of Observational Studies in Epidemiology) guidelines [38]. A completed checklist is provided in the Supplementary Materials.

The naturalistic sample we performed secondary analysis on was a three-year (2018 to 2021), chart review of consecutively treated youth in an exposure-based CBT treatment center embedded in a community mental health setting (*n* = 94). Treated diagnoses included DSM-5 recognized anxiety disorders, as well as OCD, trichotillomania, and tic disorders. Within this treatment setting, there are no comorbidity exclusions for program admittance other than active suicidality or homicidal ideation. Treatment is generally performed by master’s or doctorate-level clinicians under the close supervision of experts in CBT for OCD and anxiety.

Chart data included intake notes, session notes, and discharge summaries, all of which were deidentified prior to analysis. Trained coders abstracted baseline demographic (age, gender, racial identity, Medicaid status, and whether parents spoke English) and clinical characteristics (diagnoses, parental mental health history, and presence of suicidal ideation) from intake notes, treatment status from discharge summaries, and coded treatment techniques delivered in deidentified session notes (*n* = 2633 coded notes). Treatment response was operationalized in two ways: (1) full termination of treatment (i.e., conclusion of specialty anxiety treatment and graduation to no services), or (2) termination of specialty treatment (i.e., youth who completed specialty anxiety treatment, and either graduated to receive no additional services or transferred to ongoing supportive care or other therapy services). Full details about study methodology and primary outcomes from this chart review are published elsewhere [39]. The youth sample had a mean age of 12.35 (*SD* = 4.3, range = 4–22). Patients older than 18 were included in this sample, as in community mental health settings, patients occasionally continue to access services beyond this age. Sixty-four (68.1%) identified as female, 28 (29.8%) as male, 1 as non-binary, and 1 preferring to self-identify. Fifty-two participants (55.3%) identified as White, 14 (14.9%) as Black, 10 (10.6%) as Asian, 7 (7.4%) as Mixed Race, and 10 (10.6%) as Hispanic/Latine and White.

The study team extracted chart-documented variables on baseline characteristics, treatment-process events, and treatment outcomes. Baseline characteristic variables included gender, ethnicity, and Medicaid coverage. Treatment process variables reflected the resources utilized throughout care, including the proportion of sessions using case management services, the proportion of sessions using exposure techniques, and the total number of sessions delivered before discharge. Outcomes were represented as treatment termination or treatment termination and/or transfer to supportive care. Multiple variables extracted corresponded to common RCT exclusion criteria documented in our review, including suicidal ideation, ASD diagnosis, MDD diagnosis, medication use, ADHD diagnosis, and comorbid psychosis. All variables were drawn from the chart of each participant and coded as either ‘yes’ (diagnosis documented) or ‘no’ (diagnosis not documented). For analytic purposes, we created a binary exclusion variable, where participants were coded as ‘excluded’ if they met at least one RCT exclusion criterion (*n* = 50). Participants not meeting any of the identified exclusion criteria were coded as ‘not excluded’ (*n* = 44).

### 2.4. Statistical Analyses

Naturalistic sample data, including baseline characteristics, RCT exclusion status, and treatment process and outcome variables, were analyzed in IBM SPSS Statistics (Version 31). To compare dichotomous baseline characteristics between participants who would versus would not have been excluded from RCTs, we used χ^2^ tests of association. To evaluate differences in continuous treatment-process variables, we used descriptive statistics and conducted independent-samples *t*-tests between the RCT exclusion groups. We calculated effect sizes for these comparisons using Cohen’s *d*, providing a standardized measure of the observed group differences. To examine the impact of RCT exclusion status on treatment outcomes, we fit two binary logistic regression models, controlling for baseline demographic covariates. Each model included RCT exclusion status as the independent variable and either treatment termination and termination or transfer to supportive care as the dependent variable. Pre-analysis χ^2^ tests revealed that race was significantly associated with both Medicaid status (χ^2^ = 4.3, *p* = 0.038) and ethnicity (χ^2^ = 14.2, *p* < 0.001), suggesting multicollinearity that could distort model estimates; race was therefore excluded as a covariate. Final covariates included ethnicity, gender, and Medicaid status. Multicollinearity among retained predictors was further evaluated using variance inflation factors (VIF) and tolerance statistics; VIFs ranged from 1.01 to 1.06 across both models, well within acceptable thresholds (VIF < 5; tolerance > 0.20). As all predictors were binary, the assumption of linearity of the logit was not applicable and was not assessed. While covariate screening used a conventional threshold of α = 0.05, a conservative threshold of α = 0.01 was applied across all primary analyses to reduce the elevated risk of type I error associated with multiple comparisons.

## 3. Results

### 3.1. Exclusion Criteria in Recent CBT RCTs

Across the literature, RCTs frequently excluded participants based on factors such as comorbid psychiatric conditions, safety concerns, and concurrent treatment use. In order of frequency, the six most common exclusion criteria were current psychotropic medication use (66.7%), ASD diagnosis (63.0%), comorbid psychosis (60.5%), suicidal ideation (45.7%), MDD diagnosis (22.2%), and ADHD diagnosis (16.1%). In studies permitting an ASD diagnosis (*n* = 30), comorbidity was allowed if an anxiety disorder was the primary presenting concern and CBT was delivered to target that condition. In some RCTs (*n* = 23), psychotropic medication use was prohibited unless participants had maintained stable dosages for several weeks or months prior to the pretreatment assessment (see Table 1).

### 3.2. Trends in Exclusion Criteria over Time

When the data were examined within subsets of publication date, we found minimal change in identified exclusion criteria across decades, except for suicidal ideation. Fisher’s exact test revealed a significant association between study decade and the use of suicidal ideation as an exclusion criterion (*p* = 0.049, *V* = 0.23), with prevalence increasing across decades (2000–2009: 31.8% of studies; 2010–2019: 46.2%; 2020–2024: 85.7%). The breakdown of the common exclusion criteria by publication date is shown in Table 1.

### 3.3. Comparison to a Naturalistic Treatment Sample

We determined the proportion of participants in our naturalistic sample who would have met typical RCT inclusion criteria and whether RCT exclusion status was associated with certain characteristics and treatment outcomes. Racial and ethnic data were missing for one participant; all other demographic and baseline characteristics were fully reported. Data were missing for 3 participants (3%) on exposure use in sessions, case management use, and the total number of sessions attended. Analyses used available-case (listwise) deletion, resulting in varying sample sizes across models with these variables. No imputation procedures were used. Table 2 compares the baseline characteristics and treatment process variables between youth who were excluded from more than one RCT and those not excluded. We did not observe any significant differences in demographics between excluded and non-excluded youth. Youth who have been excluded from at least one RCT had a higher proportion of sessions involving case management (*M* = 26.1%, *SD* = 26.8%, *t*_(72.8)_ = −3.23, *p* = 0.002, *d* = −0.66) than those not excluded (*M* = 11.8%, *SD* = 14.1%). Excluded youth also had a significantly longer mean number of sessions attended (*M* = 37.56, *SD* = 27.78, *t*_(70.1)_ = −4.05, *p* < 0.001, *d* = −0.82) than those not excluded (*M* = 19.28, *SD* = 13.66). Table 3 presents the results of the binary logistic regression model, with odds ratios used as an index of effect size. RCT exclusion status did not relate to the likelihood of full treatment termination (i.e., ending specialty anxiety treatment and graduating to no therapy services) although trended in the direction of exclusion status being associated with lower likelihood of successful termination, (*OR* = 0.41, *p* = 0.051), with the model showing modest fit compared to the null model (Nagelkerke *R*^2^ = 0.17). Similarly, RCT exclusion status did not predict the likelihood of successfully concluding specialty anxiety treatment only, when including youth who ended treatment and may have gone on to additional non-anxiety focused therapy services (*OR* = 0.71, *p* = 0.478). This model demonstrated poor fit relative to the null model (Nagelkerke *R*^2^ = 0.07).

## 4. Discussion

CBT is a highly efficacious treatment for pediatric anxiety and related disorders, with multiple decades of controlled trials demonstrating its superiority over waitlist and attention controls, and in some cases, other psychotherapies [7,8,9,10,11,12,13]. Yet, data about the success of CBT when delivered in community settings has yielded mixed results, which some have theorized may be partly due to greater complexity and diversity of youth treated in community settings compared to those treated in closely controlled trial settings. To our knowledge, this is the first study to specifically catalogue common exclusion criteria used in pediatric anxiety CBT efficacy trials, examine how exclusion criteria have changed over time, and evaluate how this may influence treatment outcomes in a naturalistic sample. We found that the most common exclusion criteria in recently published RCTs were psychotropic medication use, ASD diagnosis, comorbid psychosis, suicidal ideation, MDD diagnosis, and comorbid ADHD, with exclusion based on suicidal ideation having possibly increased over the past two decades. In our chart review sample, most youth would have been ineligible for at least one RCT, consistent with prior work highlighting differences between youth treated in naturalistic vs. research settings [23,24]. Moreover, youth who would have been excluded from RCTs attended significantly more treatment sessions and required a higher proportion of sessions involving case management.

Findings align with and extend prior literature documenting a substantial gap between research samples and the youth treated in community settings. In our narrative review of recent RCTs, we found that the use of exclusion criteria in efficacy trials has remained relatively stable over time, except for a possible increase in exclusion due to suicidal ideation. While this trend should be interpreted cautiously in the absence of a systematic search, it may highlight an important and growing area of divergence between research and community practice. Furthermore, the observation that most participants in our naturalistic sample would have been excluded from at least one CBT efficacy trial raises questions about whether treatment-seeking populations may be clinically distinct from those represented in tightly controlled clinical trials. Finally, in our analysis of treatment processes in the community sample, we found that RCT exclusion status was associated with a 2.2-fold increase in the proportion of sessions involving case management and a 1.9-fold increase in session dosage. Exclusion status did not relate to the likelihood of full treatment termination or successfully concluding anxiety specialty treatment and transferring to supportive care only. Collectively, findings are consistent with the idea that youth who meet common exclusion criteria may benefit from standard CBT processes for anxiety and related disorders; however, such youth may also benefit from more complex, intensive, or prolonged care than what is typically represented in efficacy trials. This aligns with broader trends in the field that are moving away from singular-protocol approaches toward those that can more flexibly meet youth needs [33,34,40,41,42]. Overall, these results provide continual support for an “implementation cliff” in community mental health, in which intervention effects drop as EBTs move away from laboratory-controlled conditions [19]. While the observational nature of our study design restricts causal inferences, these results do suggest that a significant amount of youth in clinically representative conditions (1) would not meet criteria for inclusion in trials (53%), and (2) those who would have been excluded have more complicated and resource-intensive treatment courses.

In addition, the demographic characteristics of our naturalistic sample differed notably from those historically represented in RCTs. In our sample, 55.3% of clients identified as White, 14.9% as Black, 10.6% as Asian, and 7.4% as mixed race. By contrast, a systematic review of CBT efficacy trials reported that, among participants whose race is documented, nearly 75% are White [43]. However, we found no racial or ethnic differences between youth who would have been excluded versus included in RCTs (see Table 2), suggesting that RCT exclusion status was primarily driven by clinical characteristics rather than demographic factors. This suggests that underrepresentation of racial and ethnic minorities in pediatric anxiety RCTs may be primarily driven by recruitment and access barriers rather than exclusion criteria themselves. Furthermore, observed differences in treatment outcomes between those who would have or would not have been excluded from an RCT in the naturalistic sample may be more driven by clinical differences, rather than racial or ethnic identity. However, race was partially confounded with Medicaid status in this sample, which may reflect the association between race and adverse social determinants of health rooted in historical systemic inequities [44,45].

Findings point to important directions for future research. First, the results underscore the importance of pragmatic treatment trials with broader inclusion criteria, consistent with an ongoing shift in the literature toward enhancing the generalizability of trial findings to routine clinical populations [46,47]. Second, this study found that those who would have been excluded from RCTs required significantly more treatment sessions and case management, a result that requires further study. Instead of viewing this as indicative of decreased effectiveness of CBT for anxiety and related disorders (especially as RCT exclusion did not relate to the likelihood of successfully concluding specialty treatment), it may be reflective of a need for treatment adaptation or augmentation in this population. Third, the findings of this study could be used to help set expectations for what treatment progress might look like for youth and families. We found that RCT exclusion status was associated with longer duration of treatment and higher rates of case management use, findings that may be of prognostic use. Sharing the ‘RCT match status’ of their child may help families in understanding the prognosis of treatment with CBT and in shared decision-making about treatment adaptations. For example, explaining exposure therapy as something that should be completed within “12–16 weeks” (aligned with traditional RCT protocols) may give some families false hope of how soon they can expect treatment success to occur. Similarly, such information may help clinicians identify the extent to which a traditional CBT protocol may need to be adapted (e.g., incorporation of case management services, extended intervention length, needing to modify CBT content and delivery to address comorbid conditions) and develop appropriate expectations for how and when to expect to see treatment progress. Finally, our findings suggest that racial and ethnic background were not related to RCT exclusion status; yet the naturalistic treatment sample was much more racially and ethnically diverse than clinical trials in existing CBT literature [25,43]. To our knowledge, no meta-analyses have examined the racial and ethnic characteristics reported in CBT trials specifically for pediatric anxiety. Future research should examine disparities in representation within CBT trials for youth anxiety and identify the structural and cultural barriers that limit participation among minoritized youth.

Several limitations should be considered when interpreting the findings of this study. First, our review of RCT exclusion criteria did not rely on a full systematic review; instead, we relied on recently published meta-analyses (which did employ systematic review procedures) and backward citation chaining. Although this strategy captured a substantial number of efficacy trials, it may have resulted in the omission of eligible studies not represented in the selected meta-analyses. A notable limitation of backward citation chaining is that identified references will necessarily predate the seeded work. Given that half of the included meta-analyses were published in 2020, with two published in that year itself, the search strategy yielded only seven RCTs within the 2020–2024 timeframe. Due to this limited sample size and resulting low cell counts, Fisher’s exact test was employed to examine trends in exclusion criteria, a test that is inherently conservative and statistically underpowered [48]. This may account for the modest significance level and effect size (*p* = 0.049, *V* = 0.23) observed between study decade and the use of suicidal ideation as an exclusion criterion; thus, these findings should be interpreted with caution. Related, this review is best characterized as a narrative rather than a systematic review, and our literature sample should not be assumed to fully represent the complete body of RCTs of CBT for pediatric anxiety. Accordingly, our findings should be interpreted as preliminary and suggestive, rather than definitive. In addition, the clarity and specificity of exclusion criteria varied considerably across RCTs; some provided only broad descriptions (e.g., excluding youth with a “parent with a serious psychiatric disorder”), which introduced ambiguity during data extraction. Furthermore, because our sample comprises a heterogeneous set of RCTs, our method cannot determine how exclusion criteria may vary across geographic regions, participant age ranges (e.g., young children versus older adolescents), or diagnostic focus (e.g., trials targeting OCD vs. SAD). Furthermore, our review of RCTs of CBT for pediatric anxiety excluded effectiveness trials by design, which are designed to more accurately model routine clinical practice. This study sought to evaluate how the research conditions in tightly controlled trials, designed to minimize confounding variables to maximize internal validity and establish treatment efficacy, translate to treatment outcomes in a truly naturalistic clinical setting. To fully characterize the depth of any possible ‘implementation cliff,’ we restricted our focus to efficacy trials, as effectiveness studies with minimal exclusion criteria were unlikely to meaningfully inform this aim. Limitations also apply to the naturalistic chart review. The retrospective observational design of this study precludes causal interpretation of the associations reported here. Moreover, clinical and demographic variables were abstracted from medical records, which are subject to potential input errors and inconsistencies, and thus may not fully capture participants’ clinical presentations. Finally, the sample size was relatively small *(n* = 94), which has particular implications for interpreting logistic regression analyses. While the sample size met key assumptions for recommended events per variable [49,50], there may still have been reduced statistical power. The results of our logistic regression should not be viewed as definitive until replication in a future study with a larger sample size.

This study also had several notable strengths. First, our narrative review encompassed RCTs published between 2000 and 2024, providing a preliminary look at how exclusion criteria appear to have evolved over time. To our knowledge, this is also the first study to link RCT exclusion criteria to clinical outcomes in a community sample of racially, economically, and culturally diverse youth in an urban U.S. setting. Overall, findings suggest that youth who meet common CBT trial exclusion criteria may require longer and more complex treatment courses when receiving care in naturalistic settings. Future research broadening trial eligibility, particularly with respect to medication use and comorbid diagnoses, may help improve the translation of findings from CBT efficacy trials to community clinical settings. 

## Figures and Tables

**Table 1:** Common Exclusion Criteria by Trial Publication Date.

Decade (*n*)	Suicidal Ideation (%) *	Autism (%)	Depression (%)	Medication (%) ^a^	ADHD (%)	Comorbid Psychosis (%)
2000–2009 (22)	7 (31.8%)	14 (63.6%)	5 (22.7%)	14 (63.6%)	6 (27.3%)	14 (63.6%)
2010–2019 (52)	24 (46.2%)	32 (61.5%)	12 (23.08%)	34 (65.4%)	7 (13.5%)	33 (63.5%)
2020–2024 (7)	6 (85.7%)	5 (71.4%)	1 (14.3%)	4 (57.1%)	0 (0%)	2 (28.6%)

Note. Percentages reflect the proportion of studies for that decade which included the given criteria. ^a^ Trials excluded for psychotropic medication use in various ways. Most (*n* = 28) excluded for any medication use at all, while some (*n* = 23) required stable use for several weeks, medication use to be unrelated to OCD treatment (*n* = 2), or complete cessation for the inclusion in the trial (*n* = 1). * *p* < 0.05.

**Table 2:** Differences in Baseline Characteristics and Treatment Process between Excluded and Non-Excluded Participants (*n* = 94).

	Excluded from ≥1 RCT (*n* = 50)	Not Excluded (*n* = 44)	Test Statistic
Male Gender	12/50 (24.0%)	16/44 (36.4%)	χ^2^_(1)_ = 1.17
Ethnicity (Hispanic vs. non-Hispanic)	5/50 (10.0%)	5/43 (11.6%)	χ^2^_(1)_ = 0.00
Race (White vs. non-White)	28/50 (56.0%)	24/43 (55.8%)	χ^2^_(1)_ = 0.00
Race (Black vs. non-Black)	9/50 (18.0%)	5/43 (11.6%)	χ^2^_(1)_ = 0.32
Race (Asian vs. non-Asian)	6/50 (12.0%)	4/43 (9.3%)	χ^2^_(1)_ = 0.01
Enrolled in Medicaid	40/50 (80.0%)	28/44 (63.6%)	χ^2^_(1)_ = 2.37
Proportion of sessions using exposure	41% (*SD* = 26.8%)	42.3% (*SD* = 29.2%)	*t*_(85.7)_ = 0.22
Proportion of sessions using case management	26.1% (*SD* = 26.8%)	11.8% (*SD* = 14.1%)	*t*_(72.8)_ = −3.23 *^,a^
Sessions attended	37.56 (*SD* = 27.78)	19.28 (*SD* = 13.66)	*t*_(70.1)_ = −4.05 **^,a^

Note. For χ^2^ analyses, percentages reflect the proportion of participants within each group who possessed the given characteristic. Race and ethnicity data were missing for one participant. Due to missing data values, degrees of freedom were not constant across *t*-tests. ^a^ Significant Levene’s test for equality of variance; test statistic for equal variances not assumed reported. * *p* < 0.01; ** *p* < 0.001.

**Table 3:** Logistic Regression Models examining the Relationship between RCT Exclusion Status and Treatment Outcomes.

	*β*	S.E. (*β)*	Wald X^2^(1)	Odds Ratio (95% CI)	Nagelkerke *R*^2^	*p*
Full treatment termination	−0.90	0.46	3.82	0.41 (0.17, 1.00)	0.17	0.051
Specialty treatment termination (either full termination or transfer to supportive care	−0.34	0.48	0.50	0.71 (0.28, 1.83)	0.07	0.478

Note. CI = confidence interval. Covariates used in these models included ethnicity, gender, and Medicaid status.

## Data Availability

The data that support the findings of this study are available from the corresponding author, EBH, upon reasonable request. The data are not publicly available due to privacy and ethical reasons.

## Supplementary Materials

The following supporting information can be downloaded at: https://www.mdpi.com/article/10.3390/children13030413/s1, Table S1: STROBE Statement—checklist of items that should be included in reports of observational studies.

## Appendix A

**Table A1:** Characteristics of Selected RCTs.

Study	Study Design	Diagnoses	Sample Size	Age Range (yrs.)	Setting	Sessions	Exclusion Criteria
Arendt et al. (2016) [51]	WLC	Primary anxiety disorder	*n* = 109	7–16	Clinic	10	ADHD—Yes ASD—No Comorbid psychosis—Yes Depression—No Medication use—No Suicidal ideation—No
Asbrand et al. (2022) [52]	WLC	SAD	*n* = 119	9–13	Clinic	12	ADHD—No ASD—No Comorbid psychosis—No Depression—No Medication use—Yes Suicidal ideation—No
Aspvall et al. (2021) [53]	NIT	OCD	*n* = 152	8–17	Internet	14	ADHD—No ASD—Yes Comorbid psychosis—Yes Depression—No Medication use—Yes (stable use required) Suicidal ideation—Yes
Barrett et al. (2004) [54]	WLC	OCD	*n* = 77	7–17	Clinic	14	ADHD—Yes ASD—Yes Comorbid psychosis—Yes Depression—Yes Medication use—Yes (stable use required) Suicidal ideation—No
Bilek et al. (2022) [9]	H2H	Primary anxiety disorder	*n* = 102	7–17	Clinic	12	ADHD—No ASD—Yes Comorbid psychosis—Yes Depression—Yes Medication use—Yes Suicidal ideation—Yes
Bodden et al. (2008) [55]	H2H	Primary anxiety disorder	*n* = 128	8–18	Clinic	13	ADHD—Yes ASD—Yes Comorbid psychosis—Yes Depression—No Medication use—Yes Suicidal ideation—Yes
Bolton et al. (2008) [56]	WLC	OCD	*n* = 20	8–17	Clinic	12	ADHD—No ASD—Yes Comorbid psychosis—No Depression—No Medication use—Yes (no medications for OCD) Suicidal ideation—No
Bolton et al. (2011) [57]	WLC	OCD	*n* = 96	10–18	Clinic	12	ADHD—No ASD—Yes Comorbid psychosis—Yes Depression—Yes Medication use—Yes (stable use required) Suicidal ideation—Yes
Chalfant et al. (2007) [58]	WLC	Primary anxiety disorder ASD	*n* = 47	8–13	Clinic	12	ADHD—No ASD—Yes Comorbid psychosis—No Depression—No Medication use—Yes Suicidal ideation—No
Chavira et al. (2014) [59]	H2H	Primary anxiety disorder	*n* = 48	8–13	Clinic	10	ADHD—No ASD—Yes Comorbid psychosis—No Depression—Yes Medication use—Yes (stable use required) Suicidal ideation—Yes
Chiu et al. (2013) [60]	WLC	SAD SP GAD	*n* = 40	5–12	School	16	ADHD—No ASD—No Comorbid psychosis—No Depression—No Medication use—Yes (stable use required) Suicidal ideation—No
Cobham (2012) [61]	H2H	Primary anxiety disorder	*n* = 55	7–14	Clinic + home	12	ADHD—No ASD—No Comorbid psychosis—Yes Depression—No Medication use—Yes Suicidal ideation—No
Comer et al. (2017) [62]	H2H	OCD	*n* = 22	4–8	Internet	14	ADHD—No ASD—No Comorbid psychosis—No Depression—No Medication use—Yes Suicidal ideation—Yes
Conaughton et al. (2017) [63]	WLC	ASD SAD SP GAD	*n* = 42	8–12	Internet	16	ADHD—No ASD—Yes (participants excluded if no autism diagnosis) Comorbid psychosis—No Depression—No Medication use—No Suicidal ideation—No
Creswell et al. (2015) [64]	H2H	GAD SAD PD SP	*n* = 178	7–12	Clinic	10	ADHD—No ASD—Yes Comorbid psychosis—Yes Depression—No Medication use—Yes (stable use required) Suicidal ideation—No
Creswell et al. (2024) [65]	NIT	Primary anxiety disorder	*n* = 444	5–12	Internet	14	ADHD—No ASD—Yes Comorbid psychosis—No Depression—No Medication use—No Suicidal ideation—Yes
Donovan & March (2014) [66]	WLC	SP SAP GAD	*n* = 52	3–6	Internet	8	ADHD—No ASD—Yes Comorbid psychosis—No Depression—No Medication use—No Suicidal ideation—No
Esbjørn et al. (2015) [67]	External benchmark	GAD SAD SP SOP	*n* = 54	7–12	Clinic	12	ADHD—No ASD—No Comorbid psychosis—No Depression—No Medication use—No Suicidal ideation—No
Flannery-Schroeder & Kendall (2000) [68]	WLC	Primary anxiety disorder	*n* = 37	8–14	Clinic	18	ADHD—No ASD—No Comorbid psychosis—Yes Depression—No Medication use—Yes Suicidal ideation—No
Freeman et al. (2008) [69]	H2H	OCD	*n* = 42	5–8	Clinic	12	ADHD—Yes ASD—Yes Comorbid psychosis—Yes Depression—No Medication use—Yes (stable use required) Suicidal ideation—Yes
Freeman et al. (2014) [70]	H2H	OCD	*n* = 127	5–8	Clinic	14	ADHD—No ASD—Yes Comorbid psychosis—No Depression—No Medication use—Yes (stable use required) Suicidal ideation—Yes
Fujii et al. (2013) [71]	TAU	ASD Primary anxiety disorder	*n* = 12	7–11	Clinic	32	ADHD—Yes ASD—Yes (participants excluded if no autism diagnosis) Comorbid psychosis—Yes Depression—Yes Medication use—Yes (stable use required) Suicidal ideation—No
Gaesser & Karan (2017) [72]	WLC	Primary anxiety disorder	*n* = 63	10–18	Clinic	32	ADHD—No ASD—No Comorbid psychosis—No Depression—No Medication use—No Suicidal ideation—No
Garcia-Lopez et al. (2014) [73]	H2H	SAD	*n* = 52	13–18	School	12–17	ADHD—No ASD—Yes Comorbid psychosis—Yes Depression—No Medication use—No Suicidal ideation—Yes
Gil & Hernández-Guzmán (2009) [74]	WLC	SP	*n* = 17	7–12	Clinic	9	ADHD—No ASD—No Comorbid psychosis—No Depression—No Medication use—No Suicidal ideation—No
Ginsburg et al. (2012) [32]	WLC	Primary anxiety disorder	*n* = 12	14–17	School	10	ADHD—No ASD—No Comorbid psychosis—No Depression—No Medication use—Yes Suicidal ideation—Yes
Ginsburg & Drake (2002) [75]	TAU	GAD SOP SAD SP	*n* = 32	7–17	School	7–9	ADHD—No ASD—No Comorbid psychosis—No Depression—No Medication use—Yes Suicidal ideation—Yes
Hancock & Swain (2016) [76]	H2H	Primary anxiety disorder	*n* = 154	7–17	Clinic	10	ADHD—Yes ASD—Yes Comorbid psychosis—Yes Depression—Yes Medication use—Yes (stable use required) Suicidal ideation—Yes
Herbert et al. (2009) [77]	H2H	SAD	*n* = 73	12–17	Clinic	12	ADHD—No ASD—Yes Comorbid psychosis—Yes Depression—No Medication use—No Suicidal ideation—Yes
Hirshfeld-Becker et al. (2010) [78]	WLC	Primary anxiety disorder	*n* = 74	4–7	Clinic	16	ADHD—No ASD—No Comorbid psychosis—Yes Depression—No Medication use—Yes Suicidal ideation—Yes
Hollmann et al. (2022) [79]	WLC	OCD	*n* = 60	6–18	Internet	14	ADHD—No ASD—No Comorbid psychosis—No Depression—No Medication use—No Suicidal ideation—Yes
Hudson et al. (2009) [80]	H2H	Primary anxiety disorder	*n* = 112	7–16	Clinic	10	ADHD—No ASD—No Comorbid psychosis—Yes Depression—No Medication use—No Suicidal ideation—No
Ishikawa et al. (2019) [81]	WLC	Primary anxiety disorder	*n* = 51	8–15	Clinic	8	ADHD—No ASD—Yes Comorbid psychosis—Yes Depression—No Medication use—Yes (cessation required) Suicidal ideation—No
Kendall et al. (2008) [10]	H2H	Primary anxiety disorder	*n* = 161	7–14	Clinic	16	ADHD—No ASD—Yes Comorbid psychosis—Yes Depression—No Medication use—Yes Suicidal ideation—No
Khanna & Kendall (2010) [82]	H2H	Primary anxiety disorder	*n* = 49	7–13	Clinic	12	ADHD—No ASD—No Comorbid psychosis—Yes Depression—No Medication use—Yes Suicidal ideation—No
Lenhard et al. (2017) [83]	WLC	OCD	*n* = 67	12–17	Clinic	12	ADHD—No ASD—Yes Comorbid psychosis—Yes Depression—No Medication use—Yes (stable use required) Suicidal ideation—Yes
Leutgeb et al. (2012) [84]	WLC	Spider Phobia	*n* = 32	8–13	Clinic	1	ADHD—Yes ASD—Yes Comorbid psychosis—Yes Depression—Yes Medication use—No Suicidal ideation—No
Lewin et al. (2014) [85]	TAU	OCD	*n* = 31	3–8	Clinic	12	ADHD—No ASD—Yes Comorbid psychosis—No Depression—No Medication use—Yes (stable use required) Suicidal ideation—No
Masia-Warner et al. (2007) [86]	WLC	SAD	*n* = 36	14–16	School	12	ADHD—No ASD—No Comorbid psychosis—Yes Depression—No Medication use—Yes Suicidal ideation—Yes
McLellan et al. (2024) [87]	WLC	Primary anxiety disorder	*n* = 95	7–12	Internet	8	ADHD—No ASD—Yes Comorbid psychosis—No Depression—No Medication use—Yes Suicidal ideation—Yes
McNally-Keehn et al. (2013) [88]	WLC	ASD SAD SP GAD	*n* = 22	8–14	Clinic	16	ADHD—No ASD—Yes (participants excluded if no autism diagnosis) Comorbid psychosis—No Depression—No Medication use—No Suicidal ideation—No
Melfsen et al. (2011) [89]	WLC	SP	*n* = 44	8–14	Clinic	14	ADHD—No ASD—No Comorbid psychosis—Yes Depression—No Medication use—Yes Suicidal ideation—Yes
Merlo et al. (2010) [90]	H2H	OCD	*n* = 161	6–17	Clinic	14	ADHD—No ASD—Yes Comorbid psychosis—Yes Depression—No Medication use—Yes (stable use required) Suicidal ideation—No
Nauta et al. (2003) [91]	WLC	SAD SP GAD PD	*n* = 79	7–18	Clinic	12	ADHD—No ASD—No Comorbid psychosis—No Depression—No Medication use—Yes Suicidal ideation—No
Obiweluozo et al. (2021) [92]	WLC	SAD	*n* = 178	6–12	School	12	ADHD—No ASD—Yes Comorbid psychosis—No Depression—No Medication use—No Suicidal ideation—Yes
Ollendick et al. (2009) [93]	WLC	SP	*n* = 196	7–16	Clinic	1	ADHD—No ASD—Yes Comorbid psychosis—Yes Depression—Yes Medication use—Yes Suicidal ideation—No
Öst et al. (2015) [94]	WLC	SOP	*n* = 55	8–14	Clinic	12	ADHD—Yes ASD—Yes Comorbid psychosis—Yes Depression—Yes Medication use—Yes Suicidal ideation—No
Öst et al. (2001) [95]	WLC	SP	*n* = 60	7–17	Clinic	1	ADHD—Yes ASD—Yes Comorbid psychosis—Yes Depression—Yes Medication use—No Suicidal ideation—No
Peris & Piacentini (2013) [96]	TAU	OCD	*n* = 20	8–17	Clinic	12	ADHD—No ASD—Yes Comorbid psychosis—Yes Depression—Yes Medication use—No Suicidal ideation—No
Perrin et al. (2019) [97]	WLC	GAD	*n* = 40	10–18	Clinic	10	ADHD—No ASD—Yes Comorbid psychosis—No Depression—No Medication use—Yes Suicidal ideation—Yes
Piacentini et al. (2011) [98]	H2H	OCD	*n* = 71	8–17	Clinic	14	ADHD—No ASD—Yes Comorbid psychosis—Yes Depression—No Medication use—Yes Suicidal ideation—Yes
Pincus et al. (2010) [99]	WLC	PD	*n* = 13	14–17	Clinic	11	ADHD—No ASD—Yes Comorbid psychosis—Yes Depression—No Medication use—No Suicidal ideation—Yes
Reynolds et al. (2013) [100]	H2H	OCD	*n* = 50	12–17	Clinic	14	ADHD—No ASD—Yes Comorbid psychosis—Yes Depression—No Medication use—Yes (stable use required) Suicidal ideation—No
Santucci & Ehrenreich-May (2013) [101]	WLC	SAD	*n* = 29	7–12	Clinic	7	ADHD—No ASD—No Comorbid psychosis—Yes Depression—No Medication use—No Suicidal ideation—No
Schneider et al. (2011) [102]	WLC	SAD	*n* = 43	5–7	Clinic	16	ADHD—No ASD—No Comorbid psychosis—No Depression—No Medication use—Yes Suicidal ideation—No
Schneider et al. (2013) [103]	WLC	SAD	*n* = 64	8–13	Clinic	16	ADHD—No ASD—No Comorbid psychosis—No Depression—No Medication use—Yes Suicidal ideation—No
Shechner et al. (2014) [104]	H2H	SAD SP GAD	*n* = 55	6.5–18	Clinic	16	ADHD—No ASD—Yes Comorbid psychosis—Yes Depression—No Medication use—No Suicidal ideation—No
Silk et al. (2018) [8]	H2H	GAD SAD SP	*n* = 133	9–13	Clinic	14	ADHD—Yes ASD—Yes Comorbid psychosis—Yes Depression—Yes Medication use—Yes Suicidal ideation—Yes
Simons et al. (2006) [105]	H2H	OCD	*n* = 10	8–17	Clinic	15	ADHD—No ASD—Yes Comorbid psychosis—Yes Depression—No Medication use—Yes (no medications for OCD) Suicidal ideation—No
Southam-Gerow et al. (2010) [106]	TAU	GAD SAD SOP SP	*n* = 48	8–15	Clinic	16–20	ADHD—No ASD—Yes Comorbid psychosis—Yes Depression—No Medication use—No Suicidal ideation—No
Spence et al. (2000) [107]	H2H	SAD GAD SP SOP	*n* = 115	12–18	Internet & Clinic	10	ADHD—No ASD—Yes Comorbid psychosis—No Depression—No Medication use—No Suicidal ideation—Yes
Spence et al. (2011) [108]	WLC	SAD	*n* = 125	8–17	Internet	10	ADHD—No ASD—Yes Comorbid psychosis—Yes Depression—Yes Medication use—Yes Suicidal ideation—Yes
Spence et al. (2017) [109]	WLC	SP	*n* = 50	7–14	Clinic	12	ADHD—No ASD—Yes Comorbid psychosis—No Depression—Yes Medication use—Yes Suicidal ideation—No
Spence et al. (2006) [110]	WLC	Primary anxiety disorder	*n* = 72	7–14	Internet & Clinic	10	ADHD—No ASD—No Comorbid psychosis—No Depression—No Medication use—No Suicidal ideation—No
Sportel et al. (2013) [111]	H2H	SAD Test anxiety	*n* = 240	13–15	Internet & School	10–20	ADHD—No ASD—Yes Comorbid psychosis—No Depression—Yes Medication use—No Suicidal ideation—No
Stjerneklar et al. (2019) [112]	WLC	Primary anxiety disorder	*n* = 70	13–17	Internet	14	ADHD—No ASD—Yes Comorbid psychosis—Yes Depression—Yes Medication use—No Suicidal ideation—Yes
Storch et al. (2013) [113]	H2H	OCD	*n* = 47	7–17	Clinic	12	ADHD—No ASD—No Comorbid psychosis—No Depression—No Medication use—No Suicidal ideation—No
Storch et al. (2013) [114]	TAU	ASD SAD SP GAD OCD	*n* = 45	7–11	Clinic	18	ADHD—No ASD—Yes Comorbid psychosis—Yes Depression—No Medication use—Yes Suicidal ideation—Yes
Storch et al. (2011) [115]	WLC	OCD	*n* = 31	7–16	Internet	16	ADHD—No ASD—Yes Comorbid psychosis—No Depression—No Medication use—Yes (stable use required) Suicidal ideation—Yes
Storch et al. (2007) [116]	H2H	OCD	*n* = 41	7–17	Clinic	14	ADHD—No ASD—Yes Comorbid psychosis—Yes Depression—No Medication use—Yes (stable use required) Suicidal ideation—Yes
Storch et al. (2015) [117]	TAU	ASD SAD GAD OCD SP	*n* = 31	11–16	Clinic	16	ADHD—No ASD—No Comorbid psychosis—Yes Depression—No Medication use—Yes Suicidal ideation—Yes
Suveg et al. (2018) [118]	H2H	GAD SAD SOP	*n* = 92	7–12	Clinic	10	ADHD—No ASD—No Comorbid psychosis—Yes Depression—No Medication use—Yes Suicidal ideation—Yes
Thirlwall et al. (2013) [119]	H2H	GAD SAD SP SOP PD	*n* = 194	7–12	Clinic & Telehealth	4	ADHD—No ASD—Yes Comorbid psychosis—No Depression—No Medication use—Yes (stable use required) Suicidal ideation—No
Turner et al. (2014) [120]	NIT	OCD	*n* = 72	11–18	Telehealth	14	ADHD—No ASD—Yes Comorbid psychosis—Yes Depression—No Medication use—Yes (stable use required) Suicidal ideation—Yes
Vigerland et al. (2016) [121]	WLC	GAD PD SAD SP	*n* = 93	8–12	Internet	10	ADHD—Yes ASD—Yes Comorbid psychosis—Yes Depression—Yes Medication use—Yes (stable use required) Suicidal ideation—No
Walkup et al. (2008) [122]	H2H	SAD GAD SP	*n* = 488	7–17	Clinic	14	ADHD—Yes ASD—Yes Comorbid psychosis—Yes Depression—Yes Medication use—Yes Suicidal ideation—Yes
Waters et al. (2009) [123]	WLC	SP SOP GAD SAD	*n* = 60	4–8	Clinic	10	ADHD—Yes ASD—Yes Comorbid psychosis—Yes Depression—No Medication use—No Suicidal ideation—No
Williams et al. (2010) [124]	WLC	OCD	*n* = 21	9–18	Clinic	10	ADHD—No ASD—Yes Comorbid psychosis—Yes Depression—No Medication use—No Suicidal ideation—No
Wood et al. (2009) [125]	WLC	ASD OCD SAD SP	*n* = 40	7–11	Clinic	16	ADHD—No ASD—Yes (participants excluded if no autism diagnosis) Comorbid psychosis—No Depression—No Medication use—Yes (stable use required) Suicidal ideation—No
Wood et al. (2015) [126]	WLC	ASD Primary anxiety disorder	*n* = 33	11–15	Clinic	16	ADHD—No ASD—Yes (participants excluded if no autism diagnosis) Comorbid psychosis—Yes Depression—No Medication use—Yes (stable use required) Suicidal ideation—Yes
Wuthrich et al. (2012) [127]	WLC	Primary anxiety disorder	*n* = 43	14–17	Internet	12	ADHD—No ASD—No Comorbid psychosis—Yes Depression—No Medication use—Yes (stable use required) Suicidal ideation—Yes

Note. ASD = autism spectrum disorder; GAD = generalized anxiety disorder; OCD = obsessive–compulsive disorder; PD = panic disorder; SAD = social anxiety disorder; SOP = social phobia; SP = specific phobia; H2H = head-to-head (e.g., comparing one form of CBT to another active intervention); NIT = non-inferiority trial; TAU = treatment-as-usual; WLC = wait-list control.

## References

[1] Gandhi E., O’Gradey-Lee M., Jones A., Hudson J.L. (2022). Receipt of evidence-based care for children and adolescents with anxiety in Australia. Aust. N. Z. J. Psychiatry.

[2] Brent D., Emslie G., Clarke G., Wagner K.D., Asarnow J.R., Keller M., Vitiello B., Ritz L., Iyengar S., Abebe K. (2008). Switching to another SSRI or to venlafaxine with or without cognitive behavioral therapy for adolescents with SSRI-resistant depression: The TORDIA randomized controlled trial. JAMA.

[3] Hassan Kariri H.D., Almubaddel A. (2024). From theory to practice: Revealing the real-world impact of cognitive behavioral therapy in psychological disorders through a dynamic bibliometric and survey study. Heliyon.

[4] Thng C., Lim-Ashworth N., Poh B., Lim C.G. (2020). Recent developments in the intervention of specific phobia among adults: A rapid review. F1000Research.

[5] Wolraich M.L., Hagan J.F., Allan C., Chan E., Davison D., Earls M., Evans S.W., Flinn S.K., Froehlich T., Frost J. (2019). Clinical practice guideline for the diagnosis, evaluation, and treatment of attention-deficit/hyperactivity disorder in children and adolescents. Pediatrics.

[6] Walter H.J., Bukstein O.G., Abright A.R., Keable H., Ramtekkar U., Ripperger-Suhler J., Rockhill C. (2020). Clinical practice guideline for the assessment and treatment of children and adolescents with anxiety disorders. J. Am. Acad. Child Adolesc. Psychiatry.

[7] Sigurvinsdóttir A.L., Jensínudóttir K.B., Baldvinsdóttir K.D., Smárason O., Skarphedinsson G. (2020). Effectiveness of cognitive behavioral therapy (CBT) for child and adolescent anxiety disorders across different CBT modalities and comparisons: A systematic review and meta-analysis. Nord. J. Psychiatry.

[8] Silk J.S., Tan P.Z., Ladouceur C.D., Meller S., Siegle G.J., McMakin D.L., Forbes E.E., Dahl R.E., Kendall P.C., Mannarino A. (2018). A randomized clinical trial comparing individual cognitive behavioral therapy and child-centered therapy for child anxiety disorders. J. Clin. Child Adolesc. Psychol..

[9] Bilek E., Tomlinson R.C., Whiteman A.S., Johnson T.D., Benedict C., Phan K.L., Monk C.S., Fitzgerald K.D. (2022). Exposure-focused CBT outperforms relaxation-based control in an RCT of treatment for child and adolescent anxiety. J. Clin. Child Adolesc. Psychol..

[10] Kendall P.C., Hudson J.L., Gosch E., Flannery-Schroeder E., Suveg C. (2008). Cognitive-behavioral therapy for anxiety disordered youth: A randomized clinical trial evaluating child and family modalities. J. Consult. Clin. Psychol..

[11] Lyneham H.J., Rapee R.M. (2006). Evaluation of therapist-supported parent-implemented CBT for anxiety disorders in rural children. Behav. Res. Ther..

[12] Muris P., Meesters C., Van Melick M. (2002). Treatment of childhood anxiety disorders: A preliminary comparison between cognitive-behavioral group therapy and a psychological placebo intervention. J. Behav. Ther. Exp. Psychiatry.

[13] Rapee R.M., Abbott M.J., Lyneham H.J. (2006). Bibliotherapy for children with anxiety disorders using written materials for parents: A randomized controlled trial. J. Consult. Clin. Psychol..

[14] Philips B., Falkenström F. (2021). What research evidence is valid for psychotherapy research?. Front. Psychiatry.

[15] Lutz W., Schiefele A.-K., Wucherpfennig F., Rubel J., Stulz N. (2016). Clinical effectiveness of cognitive behavioral therapy for depression in routine care: A propensity score based comparison between randomized controlled trials and clinical practice. J. Affect. Disord..

[16] Weisz J.R., Sandler I.N., Durlak J.A., Anton B.S. (2005). Promoting and protecting youth mental health through evidence-based prevention and treatment. Am. Psychol..

[17] Sen A., Ryan P.B., Goldstein A., Chakrabarti S., Wang S., Koski E., Weng C. (2017). Correlating eligibility criteria generalizability and adverse events using Big Data for patients and clinical trials. Ann. N. Y. Acad. Sci..

[18] Van Spall H.G.C., Toren A., Kiss A., Fowler R.A. (2007). Eligibility criteria of randomized controlled trials published in high-impact general medical journals: A systematic sampling review. JAMA.

[19] Weisz J.R., Ng M.Y., Bearman S.K. (2014). Odd couple? Reenvisioning the relation between science and practice in the dissemination-implementation era. Clin. Psychol. Sci..

[20] Hudson J.L., Keers R., Roberts S., Coleman J.R.I., Breen G., Arendt K., Bögels S., Cooper P., Creswell C., Hartman C. (2015). Clinical predictors of response to cognitive-behavioral therapy in pediatric anxiety disorders: The Genes for Treatment (GxT) study. J. Am. Acad. Child Adolesc. Psychiatry.

[21] Van Vlierberghe L., Braet C., Goossens L., Mels S. (2009). Psychiatric disorders and symptom severity in referred versus non-referred overweight children and adolescents. Eur. Child Adolesc. Psychiatry.

[22] Dharni A., Coates D. (2018). Psychotropic medication profile in a community youth mental health service in Australia. Child. Youth Serv. Rev..

[23] Ehrenreich-May J., Southam-Gerow M.A., Hourigan S.E., Wright L.R., Pincus D.B., Weisz J.R. (2011). Characteristics of anxious and depressed youth seen in two different clinical contexts. Adm. Policy Ment. Health.

[24] Southam-Gerow M.A., Rodríguez A., Chorpita B.F., Daleiden E.L. (2012). Dissemination and implementation of evidence-based treatments for youth: Challenges and recommendations. Prof. Psychol. Res. Pract..

[25] Windsor L.C., Jemal A., Alessi E.J. (2015). Cognitive behavioral therapy: A meta-analysis of race and substance use outcomes. Cultur. Divers. Ethnic Minor. Psychol..

[26] Riner A.N., Girma S., Vudatha V., Mukhopadhyay N., Skoro N., Gal T.S., Freudenberger D.C., Herremans K.M., George T.J., Trevino J.G. (2022). Eligibility criteria perpetuate disparities in enrollment and participation of Black patients in pancreatic cancer clinical trials. J. Clin. Oncol..

[27] Durant R.W., Legedza A.T., Marcantonio E.R., Freeman M.B., Landon B.E. (2011). Willingness to participate in clinical trials among African Americans and whites previously exposed to clinical research. J. Cult. Divers..

[28] Scharff D.P., Mathews K.J., Jackson P., Hoffsuemmer J., Martin E., Edwards D. (2010). More than Tuskegee: Understanding mistrust about research participation. J. Health Care Poor Underserved.

[29] James A.C., Reardon T., Soler A., James G., Creswell C. (2020). Cognitive behavioral therapy for anxiety disorders in children and adolescents. Cochrane Database Syst. Rev..

[30] Asarnow J.R., Hughes J.L., Babeva K.N., Sugar C.A. (2017). Cognitive-behavioral family treatment for suicide attempt prevention: A randomized controlled trial. J. Am. Acad. Child Adolesc. Psychiatr..

[31] Beidel D.C., Ferrell C., Alfano C.A., Yeganeh R. (2001). The treatment of childhood social anxiety disorder. Psychiatr. Clin. N. Am..

[32] Ginsburg G.S., Becker K.D., Drazdowski T.K., Tein J.-Y. (2012). Treating anxiety disorders in inner city schools: Results from a pilot randomized controlled trial comparing CBT and usual Care. Child Youth Care Forum.

[33] Chorpita B.F., Daleiden E.L., Park A.L., Ward A.M., Levy M.C., Cromley T., Chiu A.W., Letamendi A.M., Tsai K.H., Krull J.L. (2017). Child STEPs in California: A cluster randomized effectiveness trial comparing modular treatment with community implemented treatment for youth with anxiety, depression, conduct problems, or traumatic stress. J. Consult. Clin. Psychol..

[34] Weisz J.R. (2012). Testing standard and modular designs for psychotherapy treating depression, anxiety, and conduct problems in youth. Arch. Gen. Psychiatry.

[35] Asnaani A., Benhamou K., Kaczkurkin A.N., Turk-Karan E., Foa E.B. (2020). Beyond the constraints of an RCT: Naturalistic treatment outcomes for anxiety-related disorders. Behav. Ther..

[36] Racz J.I., Bialocerkowski A., Calteaux I., Farrell L.J. (2024). Determinants of exposure therapy implementation in clinical practice for the treatment of anxiety, OCD, and PTSD: A systematic review. Clin. Child Fam. Psychol. Rev..

[37] Singal A.G., Higgins P.D.R., Waljee A.K. (2014). A primer on effectiveness and efficacy trials. Clin. Transl. Gastroenterol..

[38] von Elm E., Altman D.G., Egger M., Pocock S.J., Gøtzsche P.C., Vandenbroucke J.P. (2007). The Strengthening the Reporting of Observational Studies in Epidemiology (STROBE) statement: Guidelines for reporting observational studies. PLoS Med..

[39] Becker-Haimes E.M., Weiss M., Schaechter T., Young S., Sanchez A.L. (2025). Practice-based research examining effectiveness of exposure-based CBT for youth in a community mental health setting. J Mood Anxiety Disord.

[40] Kennedy S.M., Bilek E.L., Ehrenreich-May J. (2019). A randomized controlled pilot trial of the unified protocol for transdiagnostic treatment of emotional disorders in children. Behav. Modif..

[41] Weisz J., Bearman S.K., Santucci L.C., Jensen-Doss A. (2016). Initial test of a principle-guided approach to transdiagnostic psychotherapy with children and adolescents. J. Clin. Child Adolesc. Psychol..

[42] Weisz J.R., Bearman S.K. (2020). Principle-Guided Psychotherapy for Children and Adolescents: The FIRST Program for Behavioral and Emotional Problems.

[43] De Jesús-Romero R., Holder-Dixon A.R., Buss J.F., Lorenzo L.-L. (2023). Race, ethnicity, and other cultural background factors in trials of internet-based cognitive-behavioral therapy for depression: Systematic review. J. Med. Internet Res..

[44] Macias-Konstantopoulos W.L., Collins K.A., Diaz R., Duber H.C., Edwards C.D., Hsu A.P., Ranney M.L., Riviello R.J., Wettstein Z.S., Sachs C.J. (2023). Race, healthcare, and health disparities: A critical review and recommendations for advancing health equity. West. J. Emerg. Med..

[45] Thorpe R.J., Bruce M.A., Wilder T., Jones H.P., Thomas Tobin C., Norris K.C. (2025). Health disparities at the intersection of racism, social determinants of health, and downstream biological pathways. Int. J. Environ. Res. Public Health.

[46] McCashin D., Coyle D., O’Reilly G. (2022). Pesky gNATs for children experiencing low mood and anxiety—A pragmatic randomized controlled trial of technology-assisted CBT in primary care. Internet Interv..

[47] Zikopoulou O., Rapee R.M., Simos G. (2021). A randomized controlled trial of a cognitive behavior therapy program for children with clinical anxiety symptoms. Psychiatry Int..

[48] Crans G.G., Shuster J.J. (2008). How conservative is Fisher’s exact test? A quantitative evaluation of the two-sample comparative binomial trial. Stat. Med..

[49] Peduzzi P., Concato J., Kemper E., Holford T.R., Feinstein A.R. (1996). A simulation study of the number of events per variable in logistic regression analysis. J. Clin. Epidemiol..

[50] Vittinghoff E., McCulloch C.E. (2007). Relaxing the rule of ten events per variable in logistic and Cox regression. Am. J. Epidemiol..

[51] Arendt K., Thastum M., Hougaard E. (2016). Efficacy of a Danish version of the Cool Kids program: A randomized wait-list controlled trial. Acta Psychiatr. Scand..

[52] Asbrand J., Tuschen-Caffier B. (2022). Taking a closer look at social performance in childhood social anxiety disorder: Biopsychosocial context considerations and effects of cognitive behavior therapy. Children.

[53] Aspvall K., Andersson E., Melin K., Norlin L., Eriksson V., Vigerland S., Jolstedt M., Silverberg-Mörse M., Wallin L., Sampaio F. (2021). Effect of an internet-delivered stepped-care program vs in-person cognitive behavioral therapy on obsessive-compulsive disorder symptoms in children and adolescents: A randomized clinical trial. JAMA.

[54] Barrett P., Healy-Farrell L., March J.S. (2004). Cognitive-behavioral family treatment of childhood obsessive-compulsive disorder: A controlled trial. J. Am. Acad. Child Adolesc. Psychiatry.

[55] Bodden D.H., Bögels S.M., Nauta M.H., De Haan E., Ringrose J., Appelboom C., Brinkman A.G., Appelboom-Geerts K.C. (2008). Child versus family cognitive-behavioral therapy in clinically anxious youth: An efficacy and partial effectiveness study. J. Am. Acad. Child Adolesc. Psychiatry.

[56] Bolton D., Perrin S. (2008). Evaluation of exposure with response-prevention for obsessive compulsive disorder in childhood and adolescence. J. Behav. Ther. Exp. Psychiatr..

[57] Bolton D., Williams T., Perrin S., Atkinson L., Gallop C., Waite P., Salkovskis P. (2011). Randomized controlled trial of full and brief cognitive-behaviour therapy and wait-list for paediatric obsessive-compulsive disorder. J. Child Psych. Psychiatr..

[58] Chalfant A.M., Rapee R., Carroll L. (2007). Treating anxiety disorders in children with high functioning autism spectrum disorders: A controlled trial. J. Autism Dev. Disord..

[59] Chavira D.A., Drahota A., Garland A.F., Roesch S., Garcia M., Stein M.B. (2014). Feasibility of two modes of treatment delivery for child anxiety in primary care. Behav. Res. Ther..

[60] Chiu A.W., Langer D.A., McLeod B.D., Har K., Drahota A., Galla B.M., Jacobs J., Ifekwunigwe M., Wood J.J. (2013). Effectiveness of modular CBT for child anxiety in elementary schools. Sch. Psychol. Q..

[61] Cobham V.E. (2012). Do anxiety-disordered children need to come into the clinic for efficacious treatment?. J. Consult. Clin. Psychol..

[62] Comer J.S., Furr J.M., Kerns C.E., Miguel E., Coxe S., Elkins R.M., Carpenter A.L., Cornacchio D., Cooper-Vince C.E., DeSerisy M. (2017). Internet-delivered, family-based treatment for early-onset OCD: A pilot randomized trial. J. Consult. Clin. Psychol..

[63] Conaughton R.J., Donovan C.L., March S. (2017). Efficacy of an internet-based CBT program for children with comorbid high functioning autism spectrum disorder and anxiety: A randomized controlled trial. J. Affect. Disord..

[64] Creswell C., Cruddace S., Gerry S., Gitau R., McIntosh E., Mollison J., Murray L., Shafran R., Stein A., Violato M. (2015). Treatment of childhood anxiety disorder in the context of maternal anxiety disorder: A randomized controlled trial and economic analysis. Health Technol. Assess..

[65] Creswell C., Waite P., Cooper P.J. (2024). Digitally augmented, parent-led CBT versus treatment as usual for child anxiety problems in child mental health services in England and Northern Ireland: A pragmatic, non-inferiority, clinical effectiveness and cost-effectiveness randomised controlled trial. Lancet Psychiatry.

[66] Donovan C.L., March S. (2014). Online CBT for preschool anxiety disorders: A randomized control trial. Behav. Res. Ther..

[67] Esbjørn B.H., Reinholdt-Dunne M.L., Nielsen S.K., Smith A.C., Breinholst S., Leth I. (2015). Exploring the effect of case formulation driven CBT for children with anxiety disorders: A feasibility study. Behav. Cogn. Psychother..

[68] Flannery-Schroeder E.C., Kendall P.C. (2000). Group and individual cognitive-behavioral treatments for youth with anxiety disorders: A randomized clinical trial. Cognit. Ther. Res..

[69] Freeman J.B., Garcia A.M., Coyne L., Ale C., Przeworski A., Himle M., Compton S., Leonard H.L. (2008). Early childhood OCD: Preliminary findings from a family-based cognitive-behavioral approach. J. Am. Acad. Child Adolesc. Psychiatry.

[70] Freeman J., Sapyta J., Garcia A., Compton S., Khanna M., Flessner C., FitzGerald D., Mauro C., Dingfelder R., Benito K. (2014). Family-based treatment of early childhood obsessive-compulsive disorder: The Pediatric Obsessive-Compulsive Disorder Treatment Study for Young Children (POTS Jr). A randomized clinical trial. JAMA Psychiatry.

[71] Fujii C., Renno P., McLeod B.D., Lin C.E., Decker K., Zielinski K., Wood J.J. (2013). Intensive cognitive behavioral therapy for anxiety disorders in school-aged children with autism: A preliminary comparison with treatment-as-usual. Sch. Ment. Health.

[72] Gaesser A.H., Karan O.C. (2017). A randomized controlled comparison of emotional freedom technique and cognitive-behavioral therapy to reduce adolescent anxiety: A pilot study. J. Altern. Complement. Med..

[73] Garcia-Lopez L.J., Díaz-Castela M., Muela-Martinez J.A., Espinosa-Fernandez L. (2014). Can parent training for parents with high levels of expressed emotion have a positive effect on their child’s social anxiety improvement?. J. Anxiety Disord..

[74] Gil F., Hernández-Guzmán L. (2009). Cognitive-behavioural treatment in Mexican children with social phobia. An. Psicol..

[75] Ginsburg G.S., Drake K.L. (2002). School-based treatment for anxious African American adolescents: A controlled pilot study. J. Am. Acad. Child Adolesc. Psychiatr..

[76] Hancock K.M., Swain J., Cass A., Hainsworth R., Koo S., Dixon A. (2016). Long term follow up in children with anxiety disorders treated with acceptance and commitment therapy or cognitive behavioral therapy: Outcomes and predictors. J. Child Adolesc. Behav..

[77] Herbert J.D., Gaudiano B.A., Rheingold A.A., Moitra E., Myers V.H., Dalrymple K.L., Brandsma L.L. (2009). Cognitive behavior therapy for generalized social anxiety disorder in adolescents: A randomized controlled trial. J. Anxiety Disord..

[78] Hirshfeld-Becker D.R., Masek B., Henin A., Blakely L.R., Pollock-Wurman R.A., McQuade J., Biederman J. (2010). Cognitive behavioral therapy for 4- to 7-year-old children with anxiety disorders: A randomized clinical trial. J. Consult. Clin. Psychol..

[79] Hollmann K., Hohnecker C.S., Haigis A., Alt A.K., Kühnhausen J., Pascher A., Wörz U., App R., Lautenbacher H., Renner T.J. (2022). Internet-based cognitive behavioral therapy in children and adolescents with obsessive-compulsive disorder: A randomized controlled trial. Front. Psychiatry.

[80] Hudson J.L., Rapee R.M., Deveney C., Schniering C.A., Lyneham H.J., Bovopoulos N. (2009). Cognitive-behavioral treatment versus an active control for children and adolescents with anxiety disorders: A randomized trial. J. Am. Acad. Child Adolesc. Psychiatry.

[81] Ishikawa S.I., Kikuta K., Sakai M., Mitamura T., Motomura N., Hudson J.L. (2019). A randomized controlled trial of a bidirectional cultural adaptation of cognitive behavior therapy for children and adolescents with anxiety disorders. Behav. Res. Ther..

[82] Khanna M.S., Kendall P.C. (2010). Computer-assisted cognitive behavioral therapy for child anxiety: Results of a randomized clinical trial. J. Consult. Clin. Psychol..

[83] Lenhard F., Andersson E., Mataix-Cols D., Rück C., Vigerland S., Högström J., Hillborg M., Brander G., Ljungström M., Ljótsson B. (2017). Therapist-guided, internet-delivered cognitive-behavioral therapy for adolescents with obsessive-compulsive disorder: A randomized controlled trial. J. Am. Acad. Child Adolesc. Psychiatry.

[84] Leutgeb V., Schäfer A., Köchel A., Schienle A. (2012). Exposure therapy leads to enhanced late frontal positivity in 8- to 13-year-old spider phobic girls. Biol. Psychol..

[85] Lewin A.B., Park J.M., Jones A.M., Crawford E.A., DeNadai A.S., Menzel J., Storch E.A. (2014). Family-based exposure and response prevention therapy for preschool-aged children with obsessive-compulsive disorder: A pilot randomized controlled trial. Behav. Res. Ther..

[86] Masia-Warner C., Fisher P.H., Shrout P.E., Rathor S., Klein R.G. (2007). Treating adolescents with social anxiety disorder in school: An attention control trial. J. Child Psychol. Psychiatry.

[87] McLellan L.F., Woon S., Hudson J.L., Lyneham H.J., Karin E., Rapee R.M. (2024). Treating child anxiety using family-based internet delivered cognitive behavior therapy with brief therapist guidance: A randomized controlled trial. J. Anxiety Disord..

[88] McNally Keehn R.H., Lincoln A.J., Brown M.Z., Chavira D.A. (2013). The Coping Cat program for children with anxiety and autism spectrum disorder: A pilot randomized controlled trial. J. Autism Dev. Disord..

[89] Melfsen S., Kühnemund M., Schwieger J., Warnke A., Stadler C., Poustka F., Stangier U. (2011). Cognitive behavioral therapy of socially phobic children focusing on cognition: A randomised wait-list control study. Child Adolesc. Psychiatry Ment. Health.

[90] Merlo L.J., Storch E.A., Lehmkuhl H.D., Jacob M.L., Murphy T.K., Goodman W.K., Geffken G.R. (2010). Cognitive behavioral therapy plus motivational interviewing improves outcome for pediatric obsessive-compulsive disorder: A preliminary study. Cogn. Behav. Ther..

[91] Nauta M.H., Scholing A., Emmelkamp P.M., Minderaa R.B. (2003). Cognitive-behavioral therapy for children with anxiety disorders in a clinical setting: No additional effect of a cognitive parent training. J. Am. Acad. Child Adolesc. Psychiatr..

[92] Obiweluozo P.E., Ede M.O., Onwurah C.N., Uzodinma U.E., Dike I.C., Ejiofor J.N. (2021). Impact of cognitive behavioural play therapy on social anxiety among school children with stuttering deficit: A cluster randomised trial with three months follow-up. Medicine.

[93] Ollendick T.H., Öst L.G., Reuterskiöld L., Costa N., Cederlund R., Sirbu C., Davis T.E., Jarrett M.A. (2009). One-session treatment of specific phobias in youth: A randomized clinical trial in the United States and Sweden. J. Consult. Clin. Psychol..

[94] Öst L.G., Cederlund R., Reuterskiöld L. (2015). Behavioral treatment of social phobia in youth: Does parent education training improve the outcome?. Behav. Res. Ther..

[95] Öst L.G., Svensson L., Hellström K., Lindwall R. (2001). One-session treatment of specific phobias in youths: A randomized clinical trial. J. Consult. Clin. Psychol..

[96] Peris T.S., Piacentini J. (2013). Optimizing treatment for complex cases of childhood obsessive compulsive disorder: A preliminary trial. J. Clin. Child Adolesc. Psychol..

[97] Perrin S., Bevan D., Payne S., Bolton D. (2019). GAD-specific cognitive behavioral treatment for children and adolescents: A pilot randomized controlled trial. Cognit. Ther. Res..

[98] Piacentini J., Bergman R.L., Chang S., Langley A., Peris T., Wood J.J., McCracken J. (2011). Controlled comparison of family cognitive behavioral therapy and psychoeducation/relaxation training for child obsessive-compulsive disorder. J. Am. Acad. Child Adolesc. Psychiatry.

[99] Pincus D.B., May J.E., Whitton S.W., Mattis S.G., Barlow D.H. (2010). Cognitive-behavioral treatment of panic disorder in adolescence. J. Clin. Child Adolesc. Psychol..

[100] Reynolds S.A., Clark S., Smith H., Langdon P.E., Payne R., Bowers G., Norton E., McIlwham H. (2013). Randomized controlled trial of parent-enhanced CBT compared with individual CBT for obsessive-compulsive disorder in young people. J. Consult. Clin. Psychol..

[101] Santucci L.C., Ehrenreich-May J. (2013). A randomized controlled trial of the Child Anxiety Multi-Day Program (CAMP) for separation anxiety disorder. Child Psychiatry Hum. Dev..

[102] Schneider S., Blatter-Meunier J., Herren C., Adornetto C., In-Albon T., Lavallee K. (2011). Disorder-specific cognitive-behavioral therapy for separation anxiety disorder in young children: A randomized waiting-list-controlled trial. Psychother. Psychosom..

[103] Schneider S., Blatter-Meunier J., Herren C., In-Albon T., Adornetto C., Meyer A., Lavallee K.L. (2013). The efficacy of a family-based cognitive-behavioral treatment for separation anxiety disorder in children aged 8–13: A randomized comparison with a general anxiety program. J. Consult. Clin. Psychol..

[104] Shechner T., Rimon-Chakir A., Britton J.C., Lotan D., Apter A., Bliese P.D., Pine D.S., Bar-Haim Y. (2014). Attention bias modification treatment augmenting effects on cognitive behavioral therapy in children with anxiety: Randomized controlled trial. J. Am. Acad. Child Adolesc. Psychiatry.

[105] Simons M., Schneider S., Herpertz-Dahlmann B. (2006). Metacognitive therapy versus exposure and response prevention for pediatric obsessive-compulsive disorder. Psychother. Psychosom..

[106] Southam-Gerow M.A., Weisz J.R., Chu B.C., McLeod B.D., Gordis E.B., Connor-Smith J.K. (2010). Does cognitive behavioral therapy for youth anxiety outperform usual care in community clinics? An initial effectiveness test. J. Am. Acad. Child Adolesc. Psychiatry.

[107] Spence S.H., Donovan C., Brechman-Toussaint M. (2000). The treatment of childhood social phobia: The effectiveness of a social skills training-based, cognitive-behavioural intervention, with and without parental involvement. J. Child Psychol. Psychiatry.

[108] Spence S.H., Donovan C.L., March S., Gamble A., Anderson R.E., Prosser S., Kenardy J. (2011). A randomized controlled trial of online versus clinic-based CBT for adolescent anxiety. J. Consult. Clin. Psychol..

[109] Spence S.H., Donovan C.L., March S., Kenardy J.A., Hearn C.S. (2017). Generic versus disorder specific cognitive behavior therapy for social anxiety disorder in youth: A randomized controlled trial using internet delivery. Behav. Res. Ther..

[110] Spence S.H., Holmes J.M., March S., Lipp O.V. (2006). The feasibility and outcome of clinic plus internet delivery of cognitive-behavior therapy for childhood anxiety. J. Consult. Clin. Psychol..

[111] Sportel B.E., de Hullu E., de Jong P.J., Nauta M.H. (2013). Cognitive bias modification versus CBT in reducing adolescent social anxiety: A randomized controlled trial. PLoS ONE.

[112] Stjerneklar S., Hougaard E., McLellan L.F., Thastum M. (2019). A randomized controlled trial examining the efficacy of an internet-based cognitive behavioral therapy program for adolescents with anxiety disorders. PLoS ONE.

[113] Storch E.A., Arnold E.B., Lewin A.B., Nadeau J.M., Jones A.M., De Nadai A.S., Mutch P.J., Selles R.R., Ung D., Murphy T.K. (2013). The effect of cognitive-behavioral therapy versus treatment as usual for anxiety in children with autism spectrum disorders: A randomized, controlled trial. J. Am. Acad. Child Adolesc. Psychiatry.

[114] Storch E.A., Bussing R., Small B.J., Geffken G.R., McNamara J.P., Rahman O., Lewin A.B., Garvan C.S., Goodman W.K., Murphy T.K. (2013). Randomized, placebo-controlled trial of cognitive-behavioral therapy alone or combined with sertraline in the treatment of pediatric obsessive-compulsive disorder. Behav. Res. Ther..

[115] Storch E.A., Caporino N.E., Morgan J.R., Lewin A.B., Rojas A., Brauer L., Larson M.J., Murphy T.K. (2011). Preliminary investigation of web-camera delivered cognitive-behavioral therapy for youth with obsessive-compulsive disorder. Psychiatry Res..

[116] Storch E.A., Geffken G.R., Merlo L.J., Mann G., Duke D., Munson M., Adkins J., Grabill K.M., Murphy T.K., Goodman W.K. (2007). Family-based cognitive-behavioral therapy for pediatric obsessive-compulsive disorder: Comparison of intensive and weekly approaches. J. Am. Acad. Child Adolesc. Psychiatr..

[117] Storch E.A., Lewin A.B., Collier A.B., Arnold E., De Nadai A.S., Dane B.F., Nadeau J.M., Mutch P.J., Murphy T.K. (2015). A randomized controlled trial of cognitive-behavioral therapy versus treatment as usual for adolescents with autism spectrum disorders and comorbid anxiety. Depress. Anxiety.

[118] Suveg C., Jones A., Davis M., Jacob M.L., Morelen D., Thomassin K., Whitehead M. (2018). Emotion-focused cognitive-behavioral therapy for youth with anxiety disorders: A randomized trial. J. Abnorm. Child Psychol..

[119] Thirlwall K., Cooper P.J., Karalus J., Voysey M., Willetts L., Creswell C. (2013). Treatment of child anxiety disorders via guided parent-delivered cognitive-behavioural therapy: Randomised controlled trial. Br. J. Psychiatry.

[120] Turner C.M., Mataix-Cols D., Lovell K., Krebs G., Lang K., Byford S., Heyman I. (2014). Telephone cognitive-behavioral therapy for adolescents with obsessive-compulsive disorder: A randomized controlled non-inferiority trial. J. Am. Acad. Child Adolesc. Psychiatry.

[121] Vigerland S., Ljótsson B., Thulin U., Öst L.G., Andersson G., Serlachius E. (2016). Internet-delivered cognitive behavioral therapy for children with anxiety disorders: A randomised controlled trial. Behav. Res. Ther..

[122] Walkup J.T., Albano A.M., Piacentini J., Birmaher B., Compton S.N., Sherrill J.T., Ginsburg G.S., Rynn M.A., McCracken J., Waslick B. (2008). Cognitive behavioral therapy, sertraline, or a combination in childhood anxiety. N. Engl. J. Med..

[123] Waters A.M., Ford L.A., Wharton T.A., Cobham V.E. (2009). Cognitive-behavioural therapy for young children with anxiety disorders: Comparison of a child + parent condition versus a parent only condition. Behav. Res. Ther..

[124] Williams T.I., Salkovskis P.M., Forrester L., Turner S., White H., Allsopp M.A. (2010). A randomised controlled trial of cognitive behavioural treatment for obsessive compulsive disorder in children and adolescents. Eur. Child Adolesc. Psychiatry.

[125] Wood J.J., Drahota A., Sze K., Har K., Chiu A., Langer D.A. (2009). Cognitive behavioral therapy for anxiety in children with autism spectrum disorders: A randomized, controlled trial. J. Child Psychol. Psychiatry.

[126] Wood J.J., Ehrenreich-May J., Alessandri M., Fujii C., Renno P., Laugeson E., Piacentini J.C., De Nadai A.S., Arnold E., Lewin A.B. (2015). Cognitive behavioral therapy for early adolescents with autism spectrum disorders and clinical anxiety: A randomized, controlled trial. Behav. Ther..

[127] Wuthrich V.M., Rapee R.M., Cunningham M.J., Lyneham H.J., Hudson J.L., Schniering C.A. (2012). A randomized controlled trial of the Cool Teens CD-ROM computerized program for adolescent anxiety. J. Am. Acad. Child Adolesc. Psychiatry.

